# Supplementary material for: Draft Genome Assemblies and Annotations of Agrypnia vestita Walker, and Hesperophylax magnus Banks Reveal Substantial Repetitive Element Expansion in Tube Case-Making Caddisflies (Insecta: Trichoptera)
Source: Genome Biol Evol. 2021 Jan 27;13(3):evab013. doi: 10.1093/gbe/evab013 (PMC7936034; doi:10.1093/gbe/evab013)
Supplement: evab013_Supplementary_Data [file evab013_supplementary_data.docx]

Supplemental Material

##### **Supplementary Note 1:** DNA extraction, library preparation, and sequencing

*Agrypnia vestita*

We collected an adult *A. vestita* in Ramsey County, Roseville, Minnesota USA (45.0325°N, 93.17793°W) and immediately flash-frozen. High-molecular-weight genomic DNA was extracted using the Qiagen MagAttract HMW DNA Kit from the snap-frozen head and thorax according to the manufacturer's directions, performed at the Laboratories of Analytical Biology at the Smithsonian Institution’s National Museum of Natural History (NMNH). HMW Genomic DNA was quantified by fluorometry (Qubit, Thermo Fisher Scientific, Waltham, USA) and assessed for size by 1.0% (w/v) agarose gel using Pulse Field Electrophoresis (PFGE) agarose gel electrophoresis (Chef Mapper XA, Bio-Rad Laboratories, Inc).

Single Molecule Real Time (SMRT) bell libraries were prepared according to the "20 kb Template Preparation Using BluePippin Size-Selection System" as recommended by Pacific Biosciences (https://www.pacb.com/wp-content/uploads/2015/09/Procedure-Checklist-20-kb-Template-Preparation-Using-BluePippin-Size-Selection.pdf, Palo Alto, U.S.A). After damage-repair the libraries were size-selected on a BluePippin system (0.75% (w/v) agarose gel cassette, dye-free, S1 marker, high pass 20kb protocol) to remove library fragments smaller than 8 kb. Then libraries were recovered by PB AMPure beads, quantified by the high sensitivity fluorometric assay (Qubit, Thermo Fisher Scientific, Waltham, USA) and quality assessed using the genomic assay on the TapeStation (Agilent, Waldbronn, Germany). SMRT bell templates were bound to P6 polymerase using the DNA polymerase binding kit P6 v2 primers. Polymerase-template complexes were bound to magnetic beads using the Magbead Binding Kit and sequencing was carried out on the PacBio RS II (23 cells) sequencer with movie lengths of 360 min.

Illumina library preparation and sequencing was performed by Novogene using Nextera library prep followed by 2x150 paired-end sequencing on an Illumina HiSeq 2500.

*Hesperophylax magnus*

We collected a *H. magnus* pupa in Salt Lake County, Utah, USA in the Red Butte Creek (40.774273°N 111.817617°W). We extracted genomic DNA using the Agilent DNA extraction kit and prepared libraries and sequenced them using both paired-end sequencing on an Illumina NovaSeq and on four Oxford Nanopore FLO-MIN 107 flow cells using the MinION portable DNA sequencer and the LSK-109 ligation library kit.

##### **Supplementary Note 2:** Contamination-screening using BlobTools

The final genome assemblies were screened for potential contaminations with taxon

annotated GC-coverage (TAGC) plots using BlobTools v1.0. For this purpose, all Illumina reads were mapped against the final genome assemblies using BWA-MEM

v0.7.17-r1188 [(Li, 2013)](https://www.zotero.org/google-docs/?LKMnbz). Taxonomic assignment for BlobTools was done with blastn using -task megablast and -e-value 1e-25. Contigs which had a blast hit with Chordata (179 in *H. magnus*, 300 in *A. vestita*) and Cnidaria (1,054 in *A. vestita*) were filtered out (Supplemental Figure 1 and 2). The total lengths of the contamination filtered assemblies were 1,233,588,871 bp and 1,352,945,503 bp for the *H. magnus* and *A. vestita* assembly respectively.


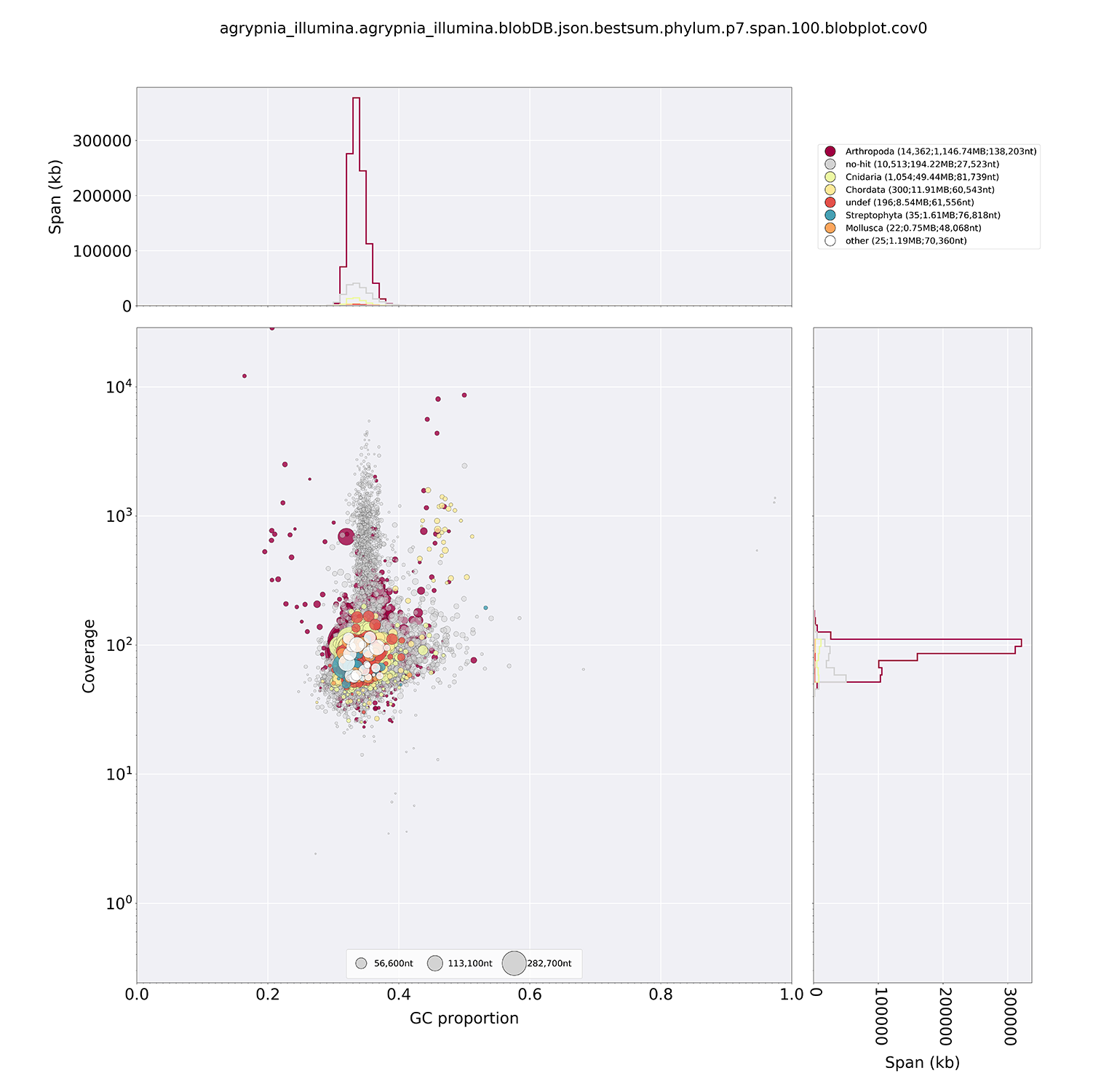


**Supplemental Figure 1.** Taxon-annotated GC-coverage (TAGC) plots for pre-filtered *A. vestita* genome assemblies*.* Circles indicate contigs and the color indicates the best match to taxon annotation. The upper and right hand panel show the total span of contigs (kb) given GC proportion.


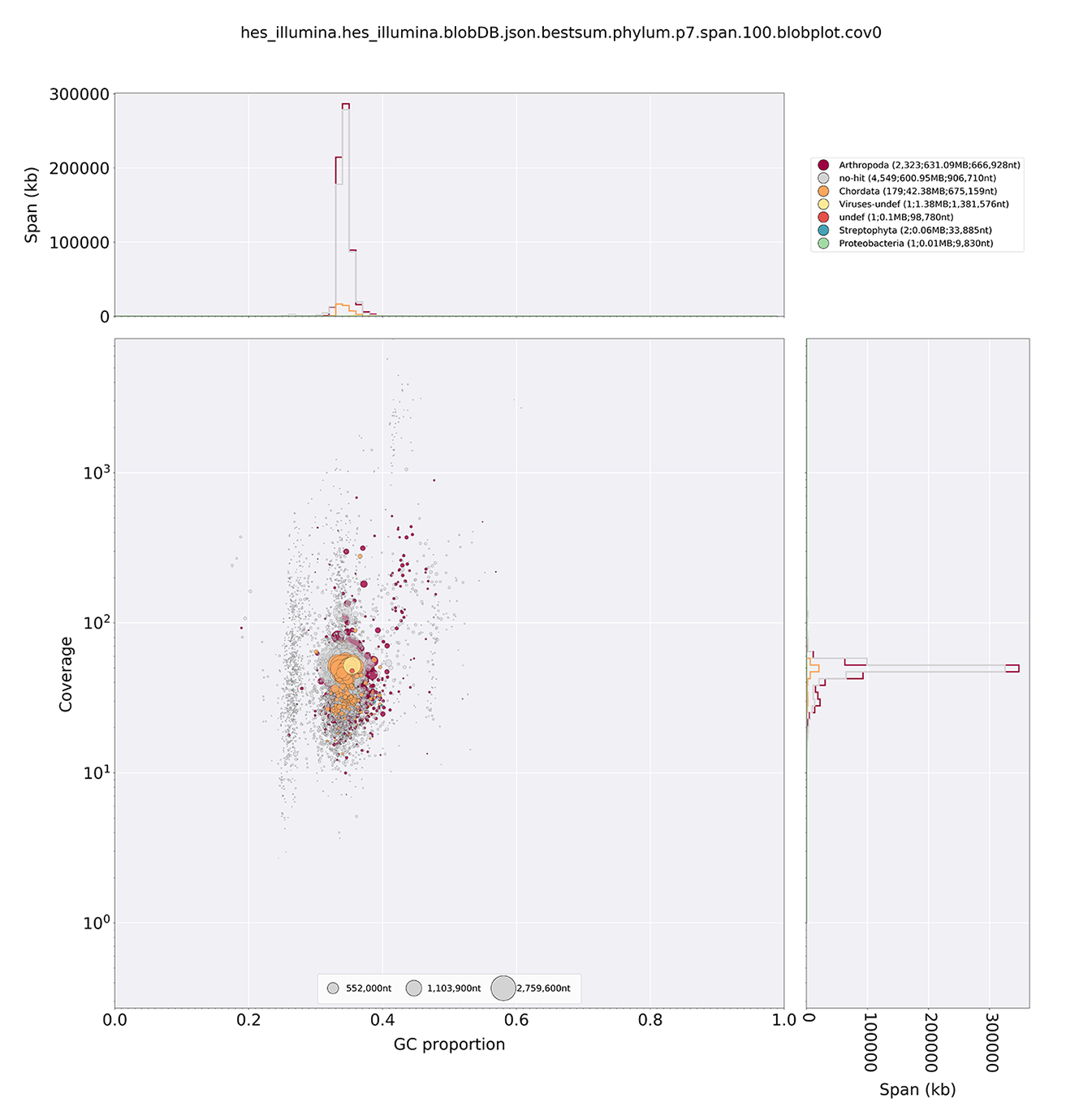


**Supplemental Figure 2.** Taxon-annotated GC-coverage (TAGC) plots for pre-filtered *H. magnus* genome assemblies*.* Circles indicate contigs and the color indicates the best match to taxon annotation. The upper and right hand panel show the total span of contigs (kb) given GC proportion.

##### **Supplementary Note 3:** BUSCO analysis

BUSCO comparisons between *Agrypnia vestita* and *Hesperophylax magnus* filtered assemblies and previous Trichoptera assemblies were done using BUSCO v4.1.1 [(Seppey et al., 2019)](https://www.zotero.org/google-docs/?BHNfMW) and the insecta_odb10 dataset (<https://busco-data.ezlab.org/v4/data/lineages/insecta_odb10.2020-08-05.tar.gz>) with options --long -m genome and --offline. The results are summarized in table 1. BUSCO v.4.1.1 was also run using the endopterygota_odb10 dataset (<https://busco-data.ezlab.org/v4/data/lineages/endopterygota_odb10.2020-08-05.tar.gz>). In total, BUSCO detected 93.3% of the Endopterygota core gene collection in the predicted proteins of *A. vestita* (complete: 88.9%, fragmented: 4.4%). For *H. magnus* 94.9% of the genes were detected (complete: 93.6%, fragmented: 1.6%).

##### **Supplementary Table 1.** Assembly statistics for filtered assemblies. Assembly stats were calculated using the assembly_stats.py function [(Mike Trizna, 2020)](https://www.zotero.org/google-docs/?5bdPvk)

| **Contig Stats** | ***A. vestita*** | ***H. magnus*** |
| --- | --- | --- |
| L10 | 267 | 26 |
| L20 | 711 | 79 |
| L30 | 1319 | 158 |
| L40 | 2134 | 262 |
| L50 | 3196 | 401 |
| N10 | 368769 | 3008714 |
| N20 | 359634 | 1845881 |
| N30 | 190978 | 1372687 |
| N40 | 14882 | 1038400 |
| N50 | 111757 | 768217 |
| GC content | 33.77 | 34.36 |
| Longest | 1130755 | 11038151 |
| Mean | 52971.52 | 179378.93 |
| Median | 25612.0 | 34916.0 |
| Sequence count | 25541 | 6877 |
| Shortest | 1057 | 1353 |
| Total bps | 1352945503 | 1233588871 |

##### **Supplementary Note 4** Genome profiling based on a k-mer distribution-based method

For genome scope profiling, Illumina reads generated in this study, as well as previously published data was used. Illumina reads of *Limnephilus lunatus* (SRR947083) and *Stenopsyche tienmushanensis* (SRR7062469) were downloaded from the European Nucleotide Archive (<https://www.ebi.ac.uk/ena>) and raw reads of *Hydropsyche tenuis* (SRS5312808) and Plectrocnemia conspersa (SRS5312807) were obtained from SRA (<https://www.ncbi.nlm.nih.gov/sra>). Raw reads were contamination filtered using kraken2 v 2.0.8-beta with the default database. Before running GenomeScope 2.0 [(Ranallo-Benavidez et al., 2020)](https://www.zotero.org/google-docs/?RaB27c), k-mers were counted with JELLYFISH v2.2.10 [(Marçais and Kingsford, 2011)](https://www.zotero.org/google-docs/?g0jpwQ) using jellyfish count -C -s 25556999998 -F 3 and a k-mer length of 21 (-m 21) as recommended for most genomes by the authors of GenomeScope with the contamination filtered Illumina reads. A histogram of k-mer frequencies was produced with jellyfish histo. GenomeScope 2.0 was run with the exported k-mer count histogram within the online web tool (http://qb.cshl.edu/genomescope/genomescope2.0/) using the following parameters:

Kmer length = 21, Max kmer coverage = 10000. Resulting GenomeScope2

profiles are available online at the links in Supplementary Table 2. We also generated genome size estimates for our two new genomes using findGSE (Sun et al., 2018), an alternative kmer based approach for estimating genome sizes. For each genome, the findGSE estimate was larger than the GenomeScope 2 estimate. While the estimates between the two approaches differed, the final assembly lengths fell in between these two estimates, suggesting that the current assembly lengths are reasonable approximations of genome size (Supplemental Fig. 2-3). Future studies using flow cytometry would help generate more precise estimates of genome sizes in caddisflies.

| **Species** | **Genome size estimate with findGSE (bp)** | **Genome size estimate with Genomescope2 (bp)** | **Unique Genomescope2 (%)** | **Link to Genomescope2 profile** |
| --- | --- | --- | --- | --- |
| *Agrypnia vestita* | 1,428,768,000 | 931,685,836 | 68.3 | http://qb.cshl.edu/genomescope/genomescope2.0/analysis.php?code=sbLuS8k74GWviwYJBMcC |
| *Hesperophylax magnus* | 1,325,002,000 | 1,060,786,686 | 57.4 | http://qb.cshl.edu/genomescope/genomescope2.0/analysis.php?code=Mt4Qb8mdRzEafda5zh0x |
| *Limnephilus lunatus* | 2,367,308,000 | 1,080,975,116 | 53.4 | http://qb.cshl.edu/genomescope/genomescope2.0/analysis.php?code=MxZkVb4nqGegS0lolPsC |
| *Stenopsyche tienmushanensis* | 498,932,000 | 389,476,263 | 85.1 | http://qb.cshl.edu/genomescope/genomescope2.0/analysis.php?code=w6vjdQTYYElr2cTsp3zK |
| *Plectrocnemia conspersa* | 460,056,000 | 316,263,089 | 98.1 | http://qb.cshl.edu/genomescope/genomescope2.0/analysis.php?code=Xuq6HXkomG412S7ksIWC |
| *Hydropsyche tenuis* | 247,424,000 | 222,816,390 | 92.6 | http://qb.cshl.edu/genomescope/genomescope2.0/analysis.php?code=swsSPZYIOejH8dfenSFm |

**Supplementary Table 2.** Genome size estimation results from both findGSE and GenomeScope 2.

##### ****

Supplemental Figure 3. Kmer profile and genome size estimates for Agrypnia vestita from findGSE (Sun et al., 2018)

##### ****

Supplemental Figure 4. Kmer profile and genome size estimates for Hesperophylax magnus from findGSE (Sun et al., 2018)

##### **Supplementary Note 5:** Repeat Analysis

We further explored repeat dynamics with a reference-free approach using RepeatExplorer2 [(Novák et al., 2013)](https://www.zotero.org/google-docs/?c1JUME) and TAREAN [(Novák et al., 2017)](https://www.zotero.org/google-docs/?4BHkeN). This orthogonal approach which estimates repeat abundance directly from short-read data can show improved quantification of repeats prone to underrepresentation in genome assemblies, such as large blocks of satellite DNA which may be present in some species. Prior to analysis with RepeatExplorer2 we normalized input reads across samples by mapping contamination filtered Illumina reads to the Endopterygota BUSCO gene set using RepeatProfiler [(Negm et al., 2020)](https://www.zotero.org/google-docs/?CZnC0s), and used average coverage values of BUSCO genes to calculate the number of reads required for 0.5x coverage of BUSCO genes per sample. To avoid bias introduced by BUSCO genes with 0 coverage genes as well as those with unexpectedly high coverage (e.g., due to unexpected mapping of repetitive sequences), we calculated 0.5x coverage using only the middle 70% of the BUSCO gene coverage values when genes were ordered by maximum coverage depth. We downsampled reads using seqtk [(Li, 2020)](https://www.zotero.org/google-docs/?iSonzE). We summarized RepeatExplorer2 output by plotting the abundance of major repeat categories present in top clusters in R (Team, R. Core).

*Results and discussion*:

A strength of RepeatExplorer2 is its ability to identify abundant satellite DNAs which can make up large fractions (e.g., >50%) of some insect genomes, and are prone to under-representation in genome assemblies. Because recombination tends to homogenize sequences within large blocks of tandem repeats, we would expect the presence of such blocks to produce superclusters which account for large fractions of the genome. However, RepeatExplorer2 clustering showed that satDNAs made up comparatively small genomic fractions in both trichopteran suborders, with genomic proportions ranging from 0.38–5.1% across study species. In addition, the distribution of superclusters and their estimated genomic proportions (see gray rectangles in Supplemental Fig. 7, especially in integripalpian species) showed that species in both suborders tend to lack superclusters of other repeat classes (*i.e*., non-satDNAs) that account for large genomic fractions. Rather, repetitive sequences group into many superclusters with each accounting for small genomic fractions, particularly in the integripalpians (Supplemental Fig. 7). This suggests that poorly represented satDNAs or other non-satDNA tandem repeats have not led to major underestimates of repetitive element content in our assemblies. This conclusion is consistent with our assembly-based repeat annotation results, which found that abundant interspersed repeats comprise the large majority or repeat content, particularly in integripalpians. Repeat classification in RepeatExplorer2 also corroborated the major findings of our assembly-based approach in that the integripalpian species showed a disproportionate increase in unclassified repeats as well as LINEs.

A difference between the assembly-based approach and RepeatExplorer2 findings is that the latter identified fewer repeats for all major repeat categories than the former, with the exception of satDNA. We suspect this is due to differences in the strengths of each approach. For example, RepeatModeler2 uses multiple independent TE identification programs in order to maximize identification of TEs in assemblies, whereas RepeatExplorer2 clustering output provides preliminary annotations of the most abundant *de novo* assembled sequences from low-coverage short-read data. In addition, the category of repeats that are most readily identified and annotated by RepeatExplorer2 (*i.e*., abundant tandem repeats) comprise small fractions of these trichopteran genomes which appear to be dominated by many variants of less-abundant interspersed repeats. Therefore, it might be expected that interspersed repeats would be underestimated by the low-coverage (*i.e*., 0.1–0.5x) assembly approach used by RepeatExplorer2. For this reason, we approached the reference-free analysis primarily as a tool to identify whether our assembly-based methods were potentially missing large blocks of abundant repeats, and not with the expectation to produce a second set of high-resolution TE classification and abundance estimates.


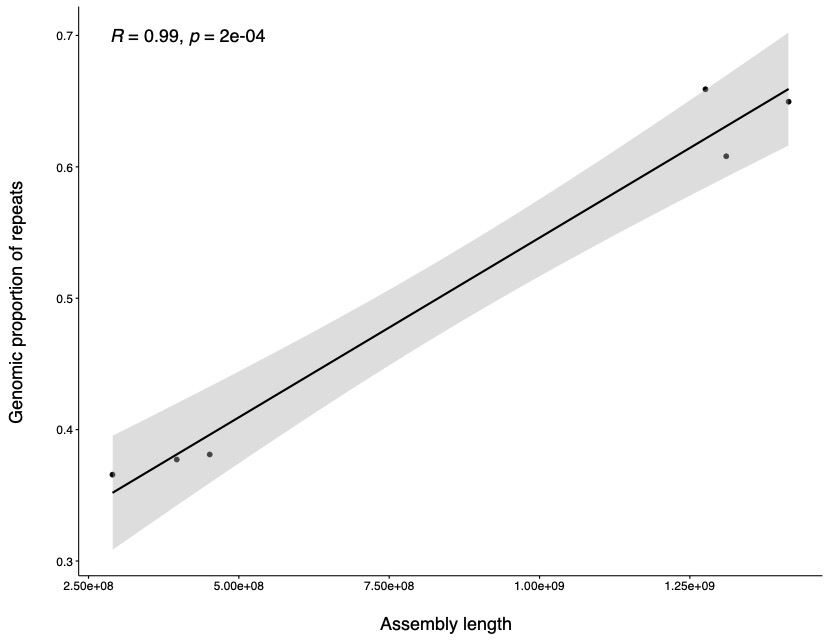


Supplemental Figure 5: Correlation between genomic repeat abundance and genome assembly length calculated using Pearson’s correlation in R.

##

##
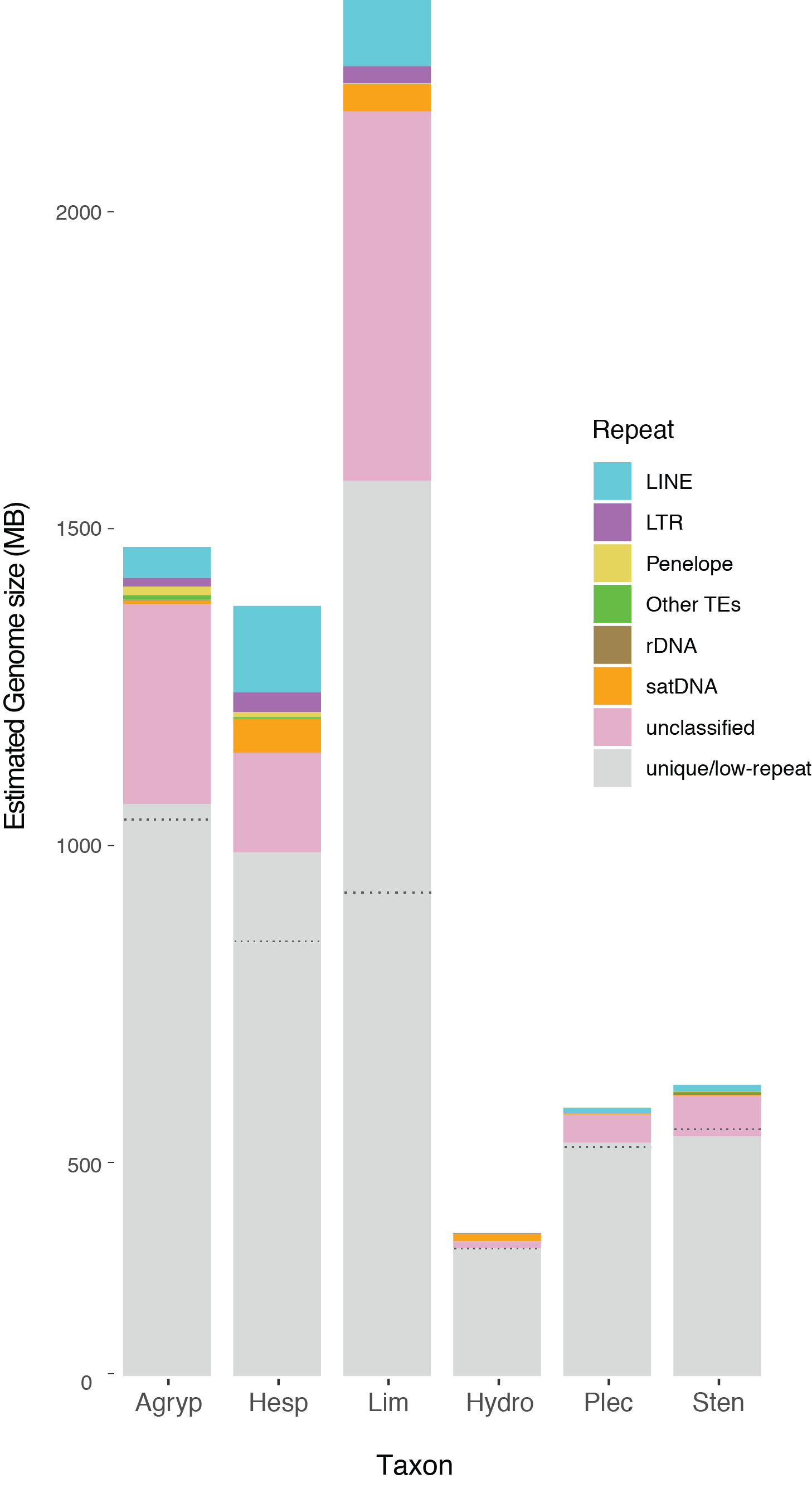


## Supplemental Figure 6: Comparison of genome size and repetitive DNA content in Trichoptera suborders estimated *de novo* from short-read sequences. Estimates include three integripalpians (Hesperophylax, Limnephilus, and Agrypnia) and three annulipalipans (Hydropsyche, Plectrocnemia, and Stenopsyche). The height of bars indicates genome size estimates obtained using findGSE and the colored segments within bars indicate the genomic proportion of major repeat categories identified by clustering analysis in RepeatExplorer2. Note that the repeat categories include annotations only from “top clusters” (represented by rectangles to the left of the dotted line in Supplemental Fig. 7), which summarize abundant repeats that were assembled and annotated from the 0.5X coverage input reads. The “unique/low-repeat” category includes both the genomic fraction of reads that were “singletons” (*i.e*., unique), and those reads that assembled as low-copy repeats but could not be assigned to a supercluster. The dotted line indicates the threshold of unique vs repetitive DNA sequences estimated by findGSE (kmer size = 21).


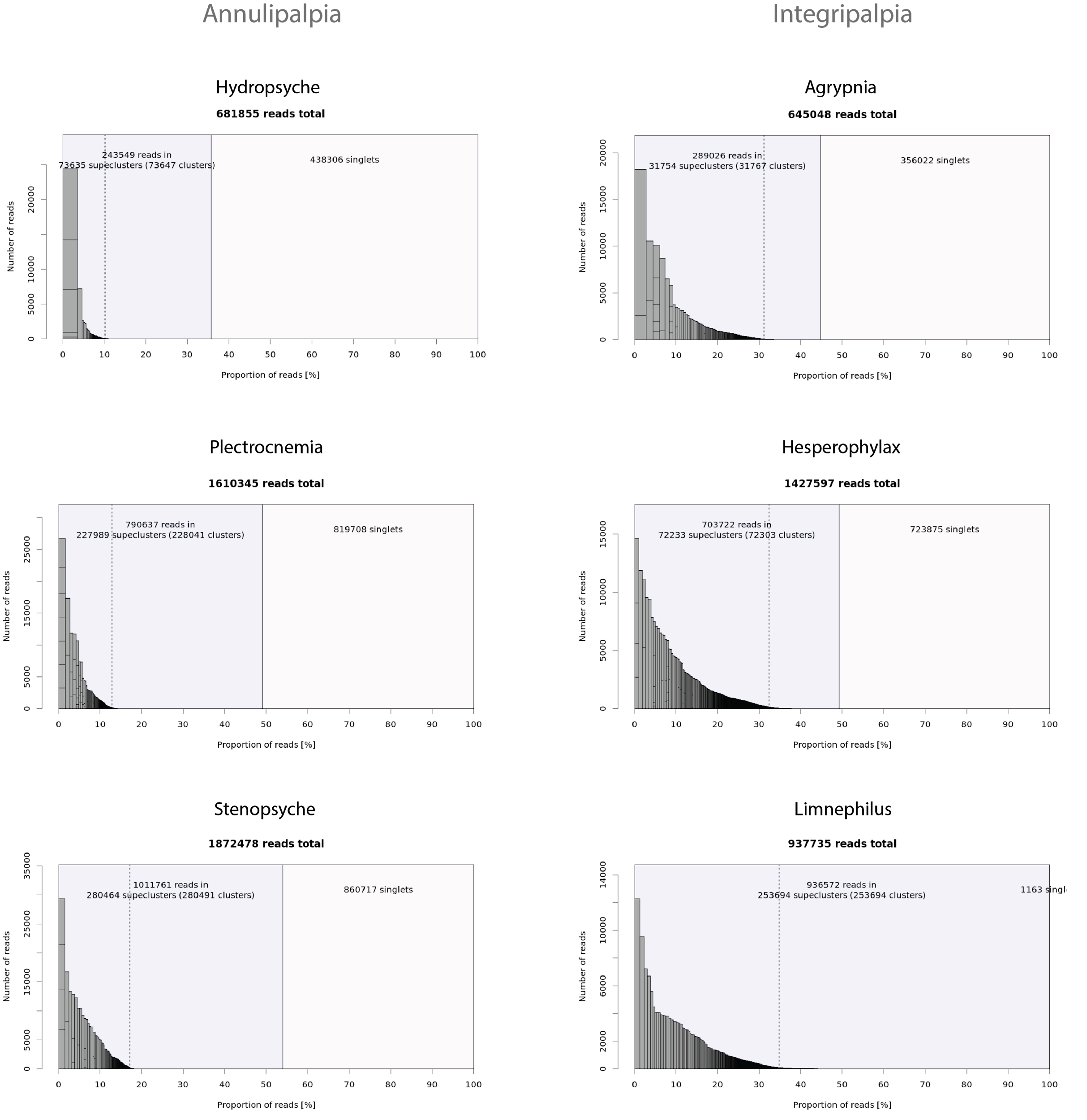


**Supplemental Figure 7:** Summary of RepeatExplorer2 clustering analysis. Gray bars represent superclusters of repetitive sequences with their heights and widths corresponding to the number of reads present in that supercluster (see y-axis). The genomic proportion of each supercluster is shown on the x-axis with top-clusters shown to the left of the dotted line. The fraction of repetitive clusters vs singlet reads is shown by the purple and pink boxes, respectively.

#####

##### **Supplementary Note 6:** Genome Annotation

Annotations for *Agrypnia vestita* and *Hesperophylax magnus* were generated using AUGUSTUS v 3.3 [(Stanke et al., 2008)](https://www.zotero.org/google-docs/?73mVc3). To generate the multiple preliminary analyses necessary for AUGUSTUS, we first ran BUSCO v.4.1.1 using the endopterygota_odb10 dataset (<https://busco-data.ezlab.org/v4/data/lineages/endopterygota_odb10.2020-08-05.tar.gz>) on the *Agrypnia vestita* and *Hesperophylax magnus* unfiltered assemblies with with options --long -m genome and --offline. Next, we ran RepeatModeler 2.0 ([Flynn et al., 2019)](https://www.zotero.org/google-docs/?SDVfdp) to identify repetitive elements. We then used RepeatMasker 4.1.0 (Smit and Hubley 2008–2015) and the output from RepeatModeler to mask all repetitive elements with hard masking turned on. We used the RepeatMasker output to create a hints file by first using the script rmOutToGFF3.pl from RepeatMasker to create a gff3 file and then using the gff2hints.pl script (<https://github.com/genomecuration/JAMg/blob/master/bin/gff2hints.pl>). Next, we aligned transcriptomes to each genome using BLAST-like Alignment Tool v3.6 (BLAT, [Kent, 2002](https://www.zotero.org/google-docs/?ZQWs6G)). We aligned the transcriptome of *P. grandis* from the 1KITE project (111126_I883_FCD0GUKACXX_L7_INShauTBBRAAPEI-22 <http://www.1kite.org/>) to *A. vestita* and the transcriptome from *H. occidentalis* [(Wang et al., 2015)](https://www.zotero.org/google-docs/?sFmJ7v) to *H. magnus* . Afterwards, we sorted the .psl file and used the script blat2hints.pl from the AUGUSTUS module. The extrinsic file contains necessary information for our sources of evidence: E (BLAT) and RM (RepeatMasker). Finally, to run AUGUSTUS we used the following input files: the assembly (hard masked), extrinsic file, merged RepeatMasker and BLAT hints files, and retrainting parameters (from BUSCO). In order to run AUGUSTUS in parallel, the script partition_EVM_inputs.pl from EVM [(Haas et al., 2008)](https://www.zotero.org/google-docs/?NYWl2W) was used to create folders with one scaffold and a corresponding hints file. At this point, scaffolds that were marked as contaminated from BlobTools were removed. The output files were then concatenated in numerical order and joined into a gff file using the script join_aug_pred.pl from AUGUSTUS. Afterwards, we characterize the gene models from AUGUSTUS using ncbi-blast2.9.0+ blastp with -e-value 1e-4, -max_hsps 5, -outfmt 5, and -max_target_seqs 10. Functional annotations were assigned using Blast2GO [(Götz et al., 2008)](https://www.zotero.org/google-docs/?3PXqXJ).

**Supplemental Figure 8:** Blast2GO Annotation Results

Pie charts showing the percentage of proteins in *A. vestita* and *H. magnus* with functional Blast2GO annotations that were verified by BLAST and mapped to GO terms compared to proteins lacking a functional annotation but verified by BLAST and mapped to GO terms or proteins only verified by BLAST.


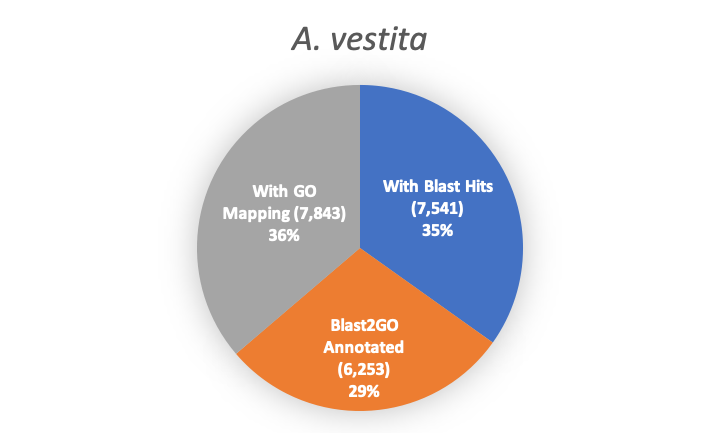


**
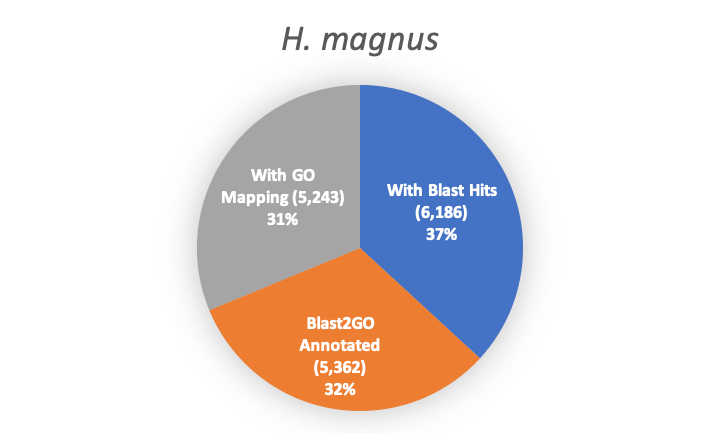
**

**
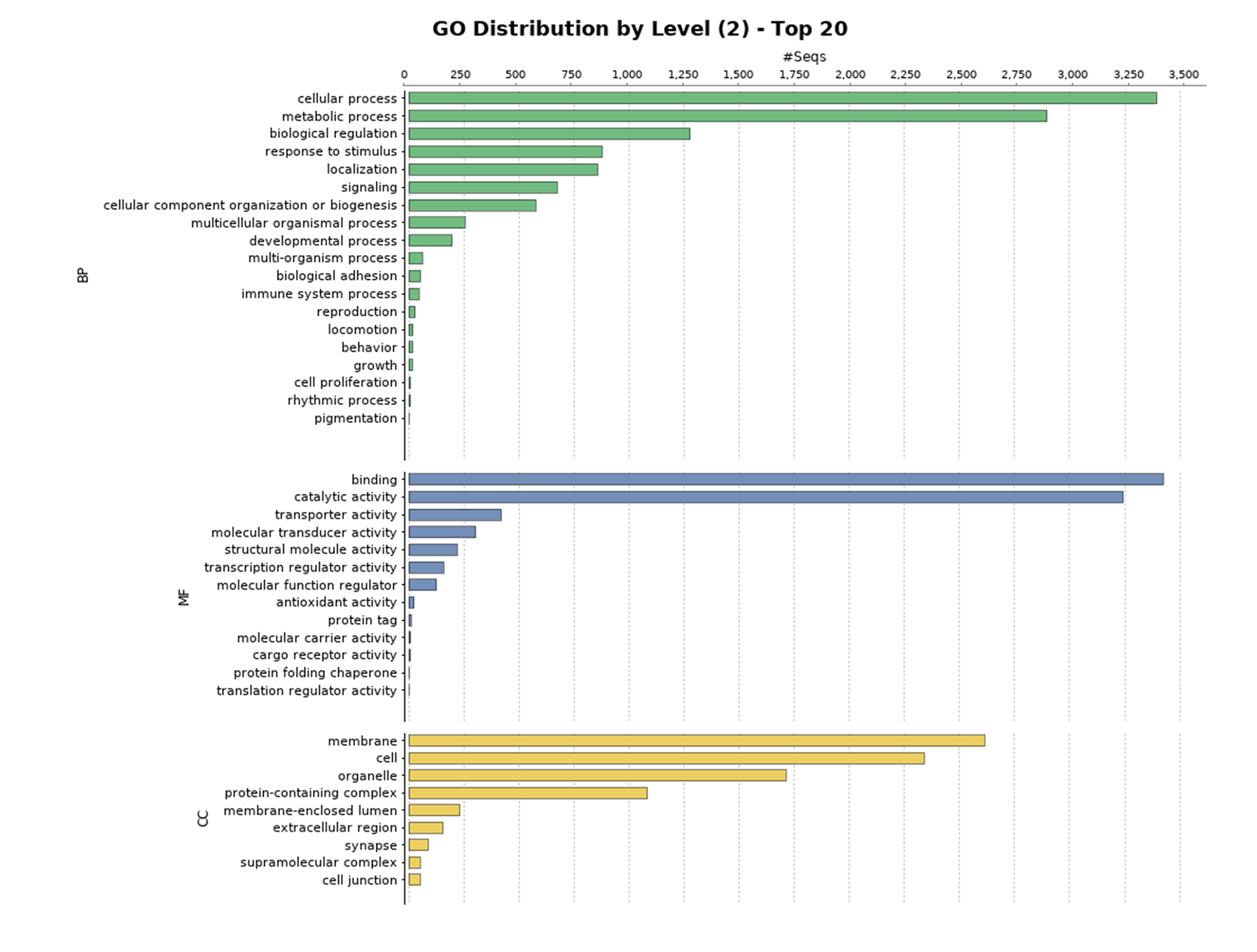
**

**Supplemental Figure 9:** Blast2GO Functional Annotation for *A. vestita*

Barplot showing GO terms characterized by biological process, molecular function, and cellular component. Barplots are grouped by biological process (BP), molecular function (MF), and cellular component (CC).

**
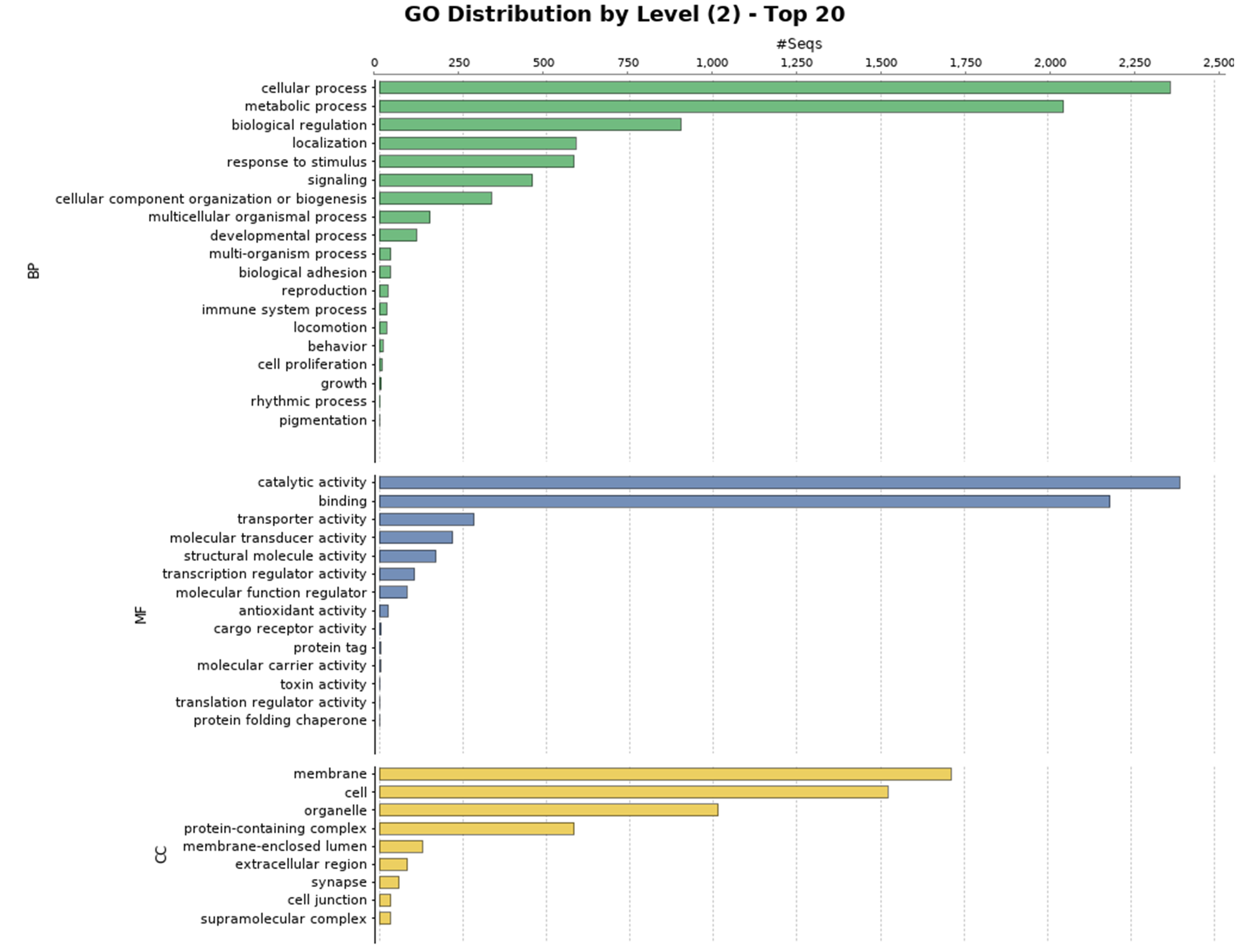
**

**Supplemental Figure 10:** Blast2GO Functional Annotation for *H. magnus*

Barplot showing GO terms characterized by biological process, molecular function, and cellular component. Barplots are grouped by biological process (BP), molecular function (MF), and cellular component (CC).

**Literature Cited**

Team, R. Core. "R: A language and environment for statistical computing." (2013).

[Flynn, J.M., Hubley, R., Goubert, C., Rosen, J., Clark, A.G., Feschotte, C., and Smit, A.F. (2019). RepeatModeler2: automated genomic discovery of transposable element families (Genomics).](https://www.zotero.org/google-docs/?oUuJtr)

[Götz, S., García-Gómez, J.M., Terol, J., Williams, T.D., Nagaraj, S.H., Nueda, M.J., Robles, M., Talón, M., Dopazo, J., and Conesa, A. (2008). High-throughput functional annotation and data mining with the Blast2GO suite. Nucleic Acids Res *36*, 3420–3435.](https://www.zotero.org/google-docs/?oUuJtr)

[Haas, B.J., Salzberg, S.L., Zhu, W., Pertea, M., Allen, J.E., Orvis, J., White, O., Buell, C.R., and Wortman, J.R. (2008). Automated eukaryotic gene structure annotation using EVidenceModeler and the Program to Assemble Spliced Alignments. Genome Biol *9*, R7.](https://www.zotero.org/google-docs/?oUuJtr)

[Kent, W.J. (2002). BLAT--the BLAST-like alignment tool. Genome Research *12*, 656–664.](https://www.zotero.org/google-docs/?oUuJtr)

[Li, H. (2013). Aligning sequence reads, clone sequences and assembly contigs with BWA-MEM. ArXiv:1303.3997 [q-Bio].](https://www.zotero.org/google-docs/?oUuJtr)

[Li, H. (2020). lh3/seqtk.](https://www.zotero.org/google-docs/?oUuJtr)

[Marçais, G., and Kingsford, C. (2011). A fast, lock-free approach for efficient parallel counting of occurrences of k-mers. Bioinformatics *27*, 764–770.](https://www.zotero.org/google-docs/?oUuJtr)

[Mike Trizna (2020). assembly_stats 0.1.4 (Zenodo).](https://www.zotero.org/google-docs/?oUuJtr)

[Negm, S., Greenberg, A., Larracuente, A.M., and Sproul, J.S. (2020). RepeatProfiler: a pipeline for visualization and comparative analysis of repetitive DNA profiles. BioRxiv 2020.05.22.111252.](https://www.zotero.org/google-docs/?oUuJtr)

[Novák, P., Neumann, P., Pech, J., Steinhaisl, J., and Macas, J. (2013). RepeatExplorer: a Galaxy-based web server for genome-wide characterization of eukaryotic repetitive elements from next-generation sequence reads. Bioinformatics *29*, 792–793.](https://www.zotero.org/google-docs/?oUuJtr)

[Novák, P., Ávila Robledillo, L., Koblížková, A., Vrbová, I., Neumann, P., and Macas, J. (2017). TAREAN: a computational tool for identification and characterization of satellite DNA from unassembled short reads. Nucleic Acids Res *45*, e111–e111.](https://www.zotero.org/google-docs/?oUuJtr)

[Ranallo-Benavidez, T.R., Jaron, KS, and Schatz, M.C. (2020). GenomeScope 2.0 and Smudgeplot for reference-free profiling of polyploid genomes. Nat Commun *11*.](https://www.zotero.org/google-docs/?oUuJtr)

[Seppey, M., Manni, M., and Zdobnov, E.M. (2019). BUSCO: Assessing Genome Assembly and Annotation Completeness. In Gene Prediction: Methods and Protocols, M. Kollmar, ed. (New York, NY: Springer), pp. 227–245.](https://www.zotero.org/google-docs/?oUuJtr)

[Stanke, M., Diekhans, M., Baertsch, R., and Haussler, D. (2008). Using native and syntenically mapped cDNA alignments to improve de novo gene finding. Bioinformatics (Oxford, England) *24*, 637–644.](https://www.zotero.org/google-docs/?oUuJtr)

Sun, H., Ding, J., Piednoël, M., Schneeberger, K. (2018). findGSE: estimating genome size variation within human and *Arabidopsis* using k-mer frequencies. Bioinformatics (Oxford, England) *34*, 550-557.

[Wang, C.-S., Pan, H., Weerasekare, G.M., and Stewart, R.J. (2015). Peroxidase-catalysed interfacial adhesion of aquatic caddisworm silk. Journal of The Royal Society Interface *12*, 20150710.](https://www.zotero.org/google-docs/?oUuJtr)
